# The Association Between Volunteering and Memory: Does Wealth Matter?

**DOI:** 10.1093/geroni/igaf122.4209

**Published:** 2025-12-31

**Authors:** Alanna Frost

**Affiliations:** University of Massachusetts Boston, Windham, New Hampshire, United States

## Abstract

**Objective:**

The benefits of volunteering and cognitive function have been explored in recent years. Although several studies have studied this relationship, no studies have examined the effect of an essential element of socioeconomic status (SES), wealth, for the relationship between volunteering and elements of cognitive function, specifically episodic memory. Therefore, the aims of this study were to investigate (1) whether volunteering is associated with memory, (2) whether wealth is related to memory, and (3) whether wealth moderates the relationship between volunteering and memory.

**Method:**

The present study utilized a nationally representative sample of 8,075 respondents aged 60 years and older, who completed both the 2020 and 2022 waves of the Health and Retirement Study.

**Results:**

Results from linear regression models revealed that older adults who volunteer have higher episodic memory scores than those who do not volunteer. Further, results indicate that higher levels of wealth are also associated with higher episodic memory scores. Results did not show a significant moderation effect of wealth for the link between volunteering and episodic memory.

**Conclusion:**

The results of this study suggest that volunteering offers cognitive benefits among older adults. Respondents who volunteer report higher levels of episodic memory compared to non-volunteers, and individuals who were wealthier also reported higher memory scores. Despite findings from the current literature suggesting that volunteering may have a greater impact on cognitive reserve among individuals of lower SES, wealth did not moderate the relationship between volunteer status and memory.

